# *N*-[(*Z*)-4-Meth­oxy­benzyl­idene](meth­oxy­carbon­yl)methanamine oxide

**DOI:** 10.1107/S1600536810032289

**Published:** 2010-08-18

**Authors:** Zeynep Keleşoğlu, Zeynep Gültekin, Orhan Büyükgüngör

**Affiliations:** a Department of Physics, Ondokuz Mayıs University, TR-55139 Samsun, Turkey; b Department of Chemistry, Çankırı Karatekin University, TR-18100 Çankırı, Turkey

## Abstract

The title compound, C_11_H_13_NO_4_, contains a nitrone group, C=N—O—*R*, the geometry of which shows a *Z* configuration with near planarity (r.m.s. deviation = 0.0787 Å) around the C=N double bond. An intra­molecular C—H⋯O hydrogen bond generates an *S*(6) ring motif. In the crystal packing, mol­ecules are linked into *R*_2_^2^(12) dimers and *R*_2_^2^(14) rings *via* C—H⋯O inter­molecular hydrogen bonds.

## Related literature

For the application and synthesis of nitro­nes, see: Merino (2004 ▶); Mocours *et al.* (1995 ▶); Frederickson (1997 ▶); Gothelf & Jorgensen (2000 ▶); Merino *et al.* (1998 ▶); McCaig *et al.* (1998 ▶); Desvergnes *et al.* (2005 ▶); Hanselmann *et al.* (2003 ▶); Pillard *et al.* (2007 ▶); Merino *et al.* (2008 ▶); Kobayashi *et al.* (2000 ▶). For the synthesis of the title compound, see: Diez-Martinez *et al.* (2010 ▶). For related structures, see: Bedford *et al.* (1991 ▶); Kliegel *et al.* (1998 ▶); Greci & Sgarabotto (1984 ▶); Christensen *et al.* (1990 ▶); Merino *et al.* (1996 ▶); Olszewski & Stadnicka (1995 ▶). For hydrogen-bond motifs, see: Bernstein *et al.* (1995 ▶).
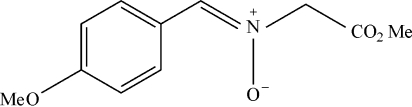


## Experimental

### 

#### Crystal data

C_11_H_13_NO_4_
*M* *_r_* = 223.22
Orthorhombic, 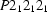

*a* = 4.3808 (3) Å
*b* = 9.8207 (7) Å
*c* = 25.7780 (17) Å
*V* = 1109.03 (13) Å^3^
*Z* = 4
Mo *K*α radiation
μ = 0.10 mm^−1^
*T* = 296 K
0.77 × 0.46 × 0.25 mm

#### Data collection

Stoe IPDS II diffractometer
Absorption correction: integration (*X-RED32*; Stoe & Cie, 2002 ▶) *T*_min_ = 0.953, *T*_max_ = 0.981
5112 measured reflections
1391 independent reflections
1053 reflections with *I* > 2σ(*I*)
*R*_int_ = 0.051

#### Refinement

*R*[*F* ^2^ > 2σ(*F* ^2^)] = 0.034
*wR*(*F* ^2^) = 0.082
*S* = 0.96
1391 reflections
146 parameters
H-atom parameters constrained
Δρ_max_ = 0.09 e Å^−3^
Δρ_min_ = −0.09 e Å^−3^

### 

Data collection: *X-AREA* (Stoe & Cie, 2002 ▶); cell refinement: *X-AREA*; data reduction: *X-RED32* (Stoe & Cie, 2002 ▶); program(s) used to solve structure: *SHELXS97* (Sheldrick, 2008 ▶); program(s) used to refine structure: *SHELXL97* (Sheldrick, 2008 ▶); molecular graphics: *ORTEP-3 for Windows* (Farrugia, 1997 ▶); software used to prepare material for publication: *WinGX* (Farrugia, 1999 ▶).

## Supplementary Material

Crystal structure: contains datablocks I, global. DOI: 10.1107/S1600536810032289/bv2158sup1.cif

Structure factors: contains datablocks I. DOI: 10.1107/S1600536810032289/bv2158Isup2.hkl

Additional supplementary materials:  crystallographic information; 3D view; checkCIF report

## Figures and Tables

**Table 1:** Hydrogen-bond geometry (Å, °)

*D*—H⋯*A*	*D*—H	H⋯*A*	*D*⋯*A*	*D*—H⋯*A*
C6—H6⋯O2	0.93	2.30	2.900 (3)	122
C8—H8⋯O3^i^	0.93	2.35	3.275 (3)	175
C9—H9*B*⋯O2^ii^	0.97	2.52	3.353 (3)	144
C11—H11*C*⋯O2^iii^	0.96	2.43	3.379 (3)	172

Symmetry codes: (i) 
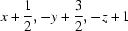
; (ii) 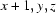; (iii) 
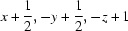
.

**Fig. 1.**
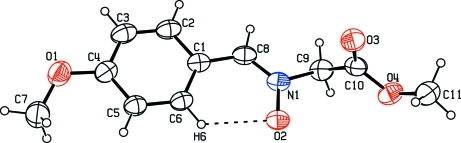
An ORTEP view of (I), with the atom-numbering scheme and 30% probability displacement ellipsoids.

**Fig. 2.**
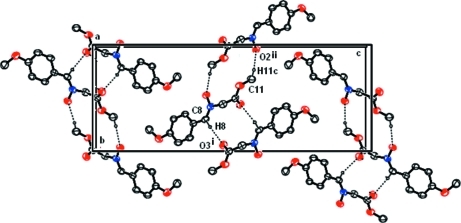
A packing diagram for (I), showing the C—H···O intermolecular hydrogen bonds, forming the R22(12) dimers and R22(14) rings. [Symmetry codes: (i) -1/2 + x, 1/2 - y, -z; (ii) -1/2 + x, -1/2 - y, -z]. H atoms not involved in hydrogen bonding (dashed lines) have been omitted for clarity.

## supplementary crystallographic information

### Comment

Nitrones are important and versatile intermediates in organic synthesis since they undergo 1,3-dipolar cycloaddition reactions with a wide range of alkynes and alkenes to afford Δ_2_-isoxazolines (Mocours *et al.*, 1995) and isoxazolidines (Gothelf & Jorgensen, 2000; Frederickson, 1997), respectively. Various cyclic or acyclic nitrones have been reported and found to be highly reactive intermediates as 1,3-dipoles (Merino, 2004). The cycloadducts have found numerous application in the synthesis of nucleosides (Merino *et al.*, 1998), amino sugars (McCaig *et al.*, 1998), pyrrollizideines (Desvergnes *et al.*, 2005) and amino acids (Hanselmann *et al.*, 2003). Nitrones are also useful intermediates for nucleophilic additions leading to the corresponding *N*,*N*-disubstituted hydroxylamines. Several types of nucleophile were used such as alkynes (Pillard *et al.*, 2007), alkyl Grignard reagent (Merino *et al.*, 2008) and enolates (Kobayashi *et al.*, 2000).

The geometry of the nitrone molecule, which shows a *Z* configuration with near planarity around the C═N double bond [C8—N1 = 1.303 (2) Å], is typical for aldonitrones (Bedford *et al.*, 1991; Kliegel *et al.*, 1998; Olszewski & Stadnicka, 1995; Greci & Sgarabotto, 1984; Christensen *et al.*, 1990). The torsion angles of O2—N1═C8—H8 and O2—N1═C8—C1 are 176.7 (3)° and -3.3 (3)°, respectively. These results are in good agreement with the literature (Merino *et al.*, 1996; Olszewski & Stadnicka, 1995). An intramolecular C6—H6···O2 hydrogen bond generates an *S*(6) ring motif (Bernstein *et al.*, 1995) (Fig. 1). In the crystal packing, molecules are linked into *R*_2_^2^(12) dimers and *R*_2_^2^(14) rings *via* C8—H8···O3 and C11—H11*C*···O2 intermolecular hydrogen bonds at (-1/2 + *x*, 1/2 - *y*, -*z*) and (-1/2 + *x*, -1/2 - *y*, -*z*), respectively (Table 1, Fig. 2). The benzene ring A (C1–C6) and *S*(6) rings are planar with the maximum r.m.s. deviation from the mean plane as -0.0234 (11) Å for N1 and these rings are coplanar with a dihedral angle of only 1.86 (10)°.

### Experimental

The title compound was synthesized by the literature method (Diez-Martinez *et al.*, 2010). To a solution of methyl glycine ester hydrochloride salt (4.0 g, 31.8 mmol) in CH_2_Cl_2_ (70 ml) was added Et_3_N (4.4 ml, 31.8 mmol) and MgSO_4_ (2 g) under an argon atmosphere at room temperature. The resulting mixture was stirred for 2 h, then anisaldehyde (3.9 ml, 31.8 mmol) was added. The reaction mixture was stirred at room temperature for an additional 24 h. The resulting precipitate was filtered through a pad of Celite and the fitrate washed with water (50 ml) and brine (50 ml), then dried over MgSO_4_. Solvent was removed under reduced pressure. The title compound imine (1) was obtained as a white solid in 77% yield. The crude imine (1) (4.0 g, 19.3 mmol) was dissolved in MeOH (50 ml) and MgSO_4_ (2 g) was added as a drying agent. To this mixture UHP (urea-hydrogen peroxide) (5.5 g, 57.9 mmol) and MeReO_3_ (methyltrioxorhenium) (96 mg, 0.38 mmol) were added under argon atmosphere at room temperature. The reaction mixture was stirred at room temperature for 7 h. After 7 h the solvent was removed *in vacuo*, the residue was washed with more CH_2_Cl_2_ (3 × 50 ml) then filtered. The filtrate removed under vacuo and the residue subjected to column chromatography eluting with EtOAc/Hexane (1:1). The title compound was obtained as a pale yellow solid in 47% yield. m.p. 65–66°C.

### Refinement

The H atoms were positioned with idealized geometry using a riding model with C—H = 0.93–0.97 Å. All H atoms were refined with isotropic displacement parameters (set to 1.2 times of the *U*_eq_ of the parent atom). 915 Friedel pairs were averaged before the final refinement as the absolute structure could not be determined unambiguously.

### Crystal data

**Table:**

C_11_H_13_NO_4_	*F*(000) = 472
*M**_r_* = 223.22	*D*_x_ = 1.337 Mg m^−^^3^
Orthorhombic, *P*2_1_2_1_2_1_	Mo *K*α radiation, λ = 0.71073 Å
Hall symbol: P 2ac 2ab	Cell parameters from 5112 reflections
*a* = 4.3808 (3) Å	θ = 2.1–27.1°
*b* = 9.8207 (7) Å	µ = 0.10 mm^−^^1^
*c* = 25.7780 (17) Å	*T* = 296 K
*V* = 1109.03 (13) Å^3^	Prism, colourless
*Z* = 4	0.77 × 0.46 × 0.25 mm

### Data collection

**Table:**

Stoe IPDS II diffractometer	1391 independent reflections
Radiation source: fine-focus sealed tube	1053 reflections with *I* > 2σ(*I*)
graphite	*R*_int_ = 0.051
rotation method scans	θ_max_ = 26.5°, θ_min_ = 2.2°
Absorption correction: integration (*X-RED32*; Stoe & Cie, 2002)	*h* = −5→5
*T*_min_ = 0.953, *T*_max_ = 0.981	*k* = −12→11
5112 measured reflections	*l* = −23→32

### Refinement

**Table:**

Refinement on *F*^2^	Secondary atom site location: difference Fourier map
Least-squares matrix: full	Hydrogen site location: inferred from neighbouring sites
*R*[*F*^2^ > 2σ(*F*^2^)] = 0.034	H-atom parameters constrained
*wR*(*F*^2^) = 0.082	*w* = 1/[σ^2^(*F*_o_^2^) + (0.0465*P*)^2^] where *P* = (*F*_o_^2^ + 2*F*_c_^2^)/3
*S* = 0.96	(Δ/σ)_max_ < 0.001
1391 reflections	Δρ_max_ = 0.09 e Å^−^^3^
146 parameters	Δρ_min_ = −0.09 e Å^−^^3^
0 restraints	Extinction correction: *SHELXL97* (Sheldrick, 2008), Fc^*^=kFc[1+0.001xFc^2^λ^3^/sin(2θ)]^-1/4^
Primary atom site location: structure-invariant direct methods	Extinction coefficient: 0.030 (4)

### Special details

**Table:**

Geometry. All e.s.d.'s (except the e.s.d. in the dihedral angle between two l.s. planes) are estimated using the full covariance matrix. The cell e.s.d.'s are taken into account individually in the estimation of e.s.d.'s in distances, angles and torsion angles; correlations between e.s.d.'s in cell parameters are only used when they are defined by crystal symmetry. An approximate (isotropic) treatment of cell e.s.d.'s is used for estimating e.s.d.'s involving l.s. planes.
Refinement. Refinement of *F*^2^ against ALL reflections. The weighted *R*-factor *wR* and goodness of fit *S* are based on *F*^2^, conventional *R*-factors *R* are based on *F*, with *F* set to zero for negative *F*^2^. The threshold expression of *F*^2^ > σ(*F*^2^) is used only for calculating *R*-factors(gt) *etc*. and is not relevant to the choice of reflections for refinement. *R*-factors based on *F*^2^ are statistically about twice as large as those based on *F*, and *R*-factors based on ALL data will be even larger.

### Fractional atomic coordinates and isotropic or equivalent isotropic displacement parameters (Å^2^)

**Table:**

	*x*	*y*	*z*	*U*_iso_*/*U*_eq_	
C1	0.6012 (4)	0.7397 (2)	0.36104 (8)	0.0608 (5)	
C2	0.5996 (5)	0.8775 (2)	0.34738 (10)	0.0734 (6)	
H2	0.7092	0.9392	0.3674	0.088*	
C3	0.4417 (6)	0.9235 (2)	0.30549 (11)	0.0808 (7)	
H3	0.4449	1.0157	0.2973	0.097*	
C4	0.2769 (5)	0.8343 (2)	0.27506 (10)	0.0700 (6)	
C5	0.2692 (5)	0.6979 (2)	0.28817 (9)	0.0669 (6)	
H5	0.1550	0.6373	0.2684	0.080*	
C6	0.4307 (5)	0.6515 (2)	0.33055 (9)	0.0642 (5)	
H6	0.4251	0.5594	0.3389	0.077*	
C7	−0.0090 (7)	0.8031 (3)	0.19685 (11)	0.0960 (8)	
H7A	−0.1660	0.7516	0.2139	0.115*	
H7B	0.1394	0.7421	0.1824	0.115*	
H7C	−0.0977	0.8568	0.1696	0.115*	
C8	0.7873 (4)	0.7021 (2)	0.40462 (9)	0.0663 (6)	
H8	0.8911	0.7726	0.4210	0.080*	
C9	1.0392 (4)	0.5582 (3)	0.46700 (10)	0.0735 (6)	
H9A	1.1358	0.6433	0.4767	0.088*	
H9B	1.1972	0.4944	0.4568	0.088*	
C10	0.8628 (4)	0.5022 (2)	0.51187 (9)	0.0651 (6)	
C11	0.8063 (7)	0.3210 (2)	0.57071 (12)	0.0936 (8)	
H11A	0.5973	0.3064	0.5610	0.112*	
H11B	0.8145	0.3798	0.6004	0.112*	
H11C	0.8999	0.2354	0.5790	0.112*	
N1	0.8278 (3)	0.58064 (18)	0.42386 (7)	0.0635 (5)	
O1	0.1341 (5)	0.88972 (18)	0.23310 (7)	0.0935 (5)	
O2	0.6925 (3)	0.47139 (14)	0.40781 (7)	0.0756 (4)	
O3	0.6482 (4)	0.55930 (17)	0.53126 (7)	0.0837 (5)	
O4	0.9683 (3)	0.38384 (16)	0.52795 (7)	0.0818 (5)	

### Atomic displacement parameters (Å^2^)

**Table:**

	*U*^11^	*U*^22^	*U*^33^	*U*^12^	*U*^13^	*U*^23^
C1	0.0565 (10)	0.0555 (12)	0.0703 (14)	−0.0055 (9)	0.0131 (10)	−0.0071 (10)
C2	0.0762 (13)	0.0549 (13)	0.0892 (17)	−0.0113 (11)	0.0137 (13)	−0.0082 (12)
C3	0.0940 (15)	0.0523 (13)	0.0962 (18)	−0.0079 (12)	0.0194 (15)	−0.0010 (13)
C4	0.0741 (12)	0.0629 (14)	0.0728 (15)	0.0046 (11)	0.0182 (11)	0.0054 (11)
C5	0.0696 (12)	0.0591 (12)	0.0720 (16)	−0.0034 (10)	0.0081 (11)	−0.0023 (10)
C6	0.0685 (11)	0.0501 (11)	0.0741 (14)	−0.0057 (9)	0.0072 (11)	−0.0025 (10)
C7	0.1023 (18)	0.098 (2)	0.0877 (18)	0.0114 (18)	−0.0029 (17)	0.0066 (15)
C8	0.0579 (10)	0.0614 (12)	0.0794 (15)	−0.0110 (10)	0.0088 (11)	−0.0134 (11)
C9	0.0527 (9)	0.0765 (15)	0.0913 (16)	0.0053 (10)	−0.0047 (12)	−0.0109 (12)
C10	0.0557 (9)	0.0672 (13)	0.0725 (14)	0.0086 (10)	−0.0106 (10)	−0.0172 (11)
C11	0.1051 (17)	0.0735 (16)	0.102 (2)	0.0058 (15)	−0.0075 (17)	0.0015 (14)
N1	0.0534 (7)	0.0632 (11)	0.0740 (12)	−0.0055 (8)	0.0053 (8)	−0.0128 (9)
O1	0.1121 (12)	0.0784 (11)	0.0898 (12)	0.0083 (11)	0.0037 (11)	0.0148 (10)
O2	0.0860 (9)	0.0599 (9)	0.0809 (10)	−0.0109 (8)	−0.0067 (9)	−0.0087 (7)
O3	0.0827 (9)	0.0877 (11)	0.0806 (11)	0.0308 (9)	0.0081 (9)	−0.0077 (9)
O4	0.0730 (8)	0.0676 (10)	0.1048 (12)	0.0181 (8)	0.0022 (9)	−0.0034 (9)

### Geometric parameters (Å, °)

**Table:**

C1—C6	1.388 (3)	C7—H7C	0.9600
C1—C2	1.399 (3)	C8—N1	1.304 (3)
C1—C8	1.436 (3)	C8—H8	0.9300
C2—C3	1.359 (4)	C9—N1	1.464 (3)
C2—H2	0.9300	C9—C10	1.496 (3)
C3—C4	1.380 (3)	C9—H9A	0.9700
C3—H3	0.9300	C9—H9B	0.9700
C4—O1	1.363 (3)	C10—O3	1.203 (2)
C4—C5	1.382 (3)	C10—O4	1.318 (3)
C5—C6	1.379 (3)	C11—O4	1.449 (3)
C5—H5	0.9300	C11—H11A	0.9600
C6—H6	0.9300	C11—H11B	0.9600
C7—O1	1.410 (3)	C11—H11C	0.9600
C7—H7A	0.9600	N1—O2	1.294 (2)
C7—H7B	0.9600		
			
C6—C1—C2	117.3 (2)	N1—C8—C1	127.57 (19)
C6—C1—C8	126.0 (2)	N1—C8—H8	116.2
C2—C1—C8	116.64 (19)	C1—C8—H8	116.2
C3—C2—C1	121.6 (2)	N1—C9—C10	108.42 (15)
C3—C2—H2	119.2	N1—C9—H9A	110.0
C1—C2—H2	119.2	C10—C9—H9A	110.0
C2—C3—C4	120.5 (2)	N1—C9—H9B	110.0
C2—C3—H3	119.8	C10—C9—H9B	110.0
C4—C3—H3	119.8	H9A—C9—H9B	108.4
O1—C4—C5	124.8 (2)	O3—C10—O4	123.7 (2)
O1—C4—C3	116.0 (2)	O3—C10—C9	123.6 (2)
C5—C4—C3	119.3 (2)	O4—C10—C9	112.71 (18)
C4—C5—C6	120.1 (2)	O4—C11—H11A	109.5
C4—C5—H5	120.0	O4—C11—H11B	109.5
C6—C5—H5	120.0	H11A—C11—H11B	109.5
C5—C6—C1	121.3 (2)	O4—C11—H11C	109.5
C5—C6—H6	119.4	H11A—C11—H11C	109.5
C1—C6—H6	119.4	H11B—C11—H11C	109.5
O1—C7—H7A	109.5	O2—N1—C8	125.08 (18)
O1—C7—H7B	109.5	O2—N1—C9	114.05 (17)
H7A—C7—H7B	109.5	C8—N1—C9	120.86 (17)
O1—C7—H7C	109.5	C4—O1—C7	119.3 (2)
H7A—C7—H7C	109.5	C10—O4—C11	116.31 (17)
H7B—C7—H7C	109.5		
			
C6—C1—C2—C3	−1.0 (3)	C2—C1—C8—N1	−179.0 (2)
C8—C1—C2—C3	177.2 (2)	N1—C9—C10—O3	−57.6 (3)
C1—C2—C3—C4	0.1 (3)	N1—C9—C10—O4	122.64 (18)
C2—C3—C4—O1	−178.1 (2)	C1—C8—N1—O2	−3.3 (3)
C2—C3—C4—C5	1.2 (3)	C1—C8—N1—C9	176.68 (18)
O1—C4—C5—C6	177.8 (2)	C10—C9—N1—O2	−60.8 (2)
C3—C4—C5—C6	−1.5 (3)	C10—C9—N1—C8	119.2 (2)
C4—C5—C6—C1	0.5 (3)	C5—C4—O1—C7	−6.6 (3)
C2—C1—C6—C5	0.7 (3)	C3—C4—O1—C7	172.7 (2)
C8—C1—C6—C5	−177.30 (19)	O3—C10—O4—C11	1.4 (3)
C6—C1—C8—N1	−0.9 (3)	C9—C10—O4—C11	−178.8 (2)

### Hydrogen-bond geometry (Å, °)

**Table:**

*D*—H···*A*	*D*—H	H···*A*	*D*···*A*	*D*—H···*A*
C6—H6···O2	0.93	2.30	2.900 (3)	122
C8—H8···O3^i^	0.93	2.35	3.275 (3)	175
C9—H9B···O2^ii^	0.97	2.52	3.353 (3)	144
C11—H11C···O2^iii^	0.96	2.43	3.379 (3)	172

Symmetry codes: (i) *x*+1/2, −*y*+3/2, −*z*+1; (ii) *x*+1, *y*, *z*; (iii) *x*+1/2, −*y*+1/2, −*z*+1.
